# Comparison of the protein-coding gene content of *Chlamydia trachomatis* and *Protochlamydia amoebophila* using a Raspberry Pi computer

**DOI:** 10.1186/s13104-015-1476-2

**Published:** 2015-10-13

**Authors:** James F. Robson, Daniel Barker

**Affiliations:** School of Biology, University of St Andrews, St Andrews, Fife, KY16 9TH UK; Department of Biology, University of York, Wentworth Way, York, YO10 5DD UK

**Keywords:** *Chlamydia*, *Protochlamydia*, Chlamydiae, Parasite, Endosymbiont, Bioinformatics, Comparative genomics, Protein families, Raspberry Pi

## Abstract

**Background:**

To demonstrate the bioinformatics capabilities of a low-cost computer, the Raspberry Pi, we present a comparison of the protein-coding gene content of two species in phylum Chlamydiae: *Chlamydia trachomatis,* a common sexually transmitted infection of humans, and Candidatus *Protochlamydia amoebophila,* a recently discovered amoebal endosymbiont. Identifying species-specific proteins and differences in protein families could provide insights into the unique phenotypes of the two species.

**Results:**

Using a Raspberry Pi computer, sequence similarity-based protein families were predicted across the two species, *C. trachomatis* and *P. amoebophila*, and their members counted. Examples include nine multi-protein families unique to *C. trachomatis,* 132 multi-protein families unique to *P. amoebophila* and one family with multiple copies in both. Most families unique to *C. trachomatis* were polymorphic outer-membrane proteins. Additionally, multiple protein families lacking functional annotation were found. Predicted functional interactions suggest one of these families is involved with the exodeoxyribonuclease V complex.

**Conclusion:**

The Raspberry Pi computer is adequate for a comparative genomics project of this scope. The protein families unique to *P. amoebophila* may provide a basis for investigating the host-endosymbiont interaction. However, additional species should be included; and further laboratory research is required to identify the functions of unknown or putative proteins. Multiple outer membrane proteins were found in *C. trachomatis*, suggesting importance for host evasion. The tyrosine transport protein family is shared between both species, with four proteins in *C. trachomatis* and two in *P. amoebophila.* Shared protein families could provide a starting point for discovery of wide-spectrum drugs against Chlamydiae.

**Electronic supplementary material:**

The online version of this article (doi:10.1186/s13104-015-1476-2) contains supplementary material, which is available to authorized users.

## Background

The Raspberry Pi [1] is one of a recent wave of small, general-purpose computers, delivering moderate computer power at low cost and with very modest requirements for electrical power [2]. It was released by the Raspberry Pi Foundation in 2012, primarily with school-level educational in mind [3]. The various models of Raspberry Pi have now sold over 5 million units in total [4], and have found a wide range of uses in addition to those originally envisaged. For example, the Raspberry Pi is being used in university-level education in bioinformatics [5] and radiology [6], for field genomics with the portable, USB-powered Oxford Nanopore MinION sequencer [7], for eukaryotic genome assembly [8] and in clusters [9]. The Raspberry Pi may have a future role in clinical diagnosis [10]. Computers such as the Raspberry Pi could provide a cheap and reliable platform to perform powerful analysis in remote, rural or pandemic-stricken areas.

We present a preliminary comparative genomics study, carried out on the Pi as coursework for the module BL4273 Bioinformatics for Biologists at the University of St Andrews in 2014. (An Open Access version of the BL4273 teaching material has been released as part of 4273*π* [5]). Our study is limited in scope, due to use of only two species. However, it uses bioinformatics research software typical of a current study, and leads to suggestions for future research. Our study and [11] demonstrate the suitability of the Raspberry Pi for bioinformatics research in comparative genomics.

We compare the genomes of *Chlamydia trachomatis* and Candidatus *Protochlamydia amoebophila*. *Chlamydia* is a genus of obligate intracellular bacteria within the phylum Chlamydiae. Environmental Chlamydiae and the clade now consisting of human-pathogenic Chlamydiae diverged from each other around 700 million years ago [12]. Among the pathogens of humans is *C.trachomatis*. *C. trachomatis* infection is one of the most common sexually transmitted diseases and if untreated can result in trachoma, causing many ailments including blindness, pelvic inflammatory disease, chronic pelvic pain, ectopic pregnancy and epidymitis [13].

The related endosymbiont Candidatus *Protochlamydia amoebophilia* was discovered living in the amoeba *Acanthamoeba.* Among the genome of *P. amoebophilia*, genes coding for type III and IV secretion machinery have been identified, although effector molecules have yet to be found [14]. *C. trachomatis* has a reduced genome, and lacks genes for various pathways present in the human body. For example, *P. amoebophilia* has all TCA cycle genes, whereas most pathogenic *Chlamydia* lack the full metabolic pathway [12]. Additionally, pathogenic *Chlamydia* lack other metabolic and biosynthetic genes, such as some amino acid synthesis genes [15].

In this preliminary investigation of the two species, we hope to identify groups of genes which are unique to one or both species, identify function and to guide further *Chlamydia* research.

## Results

The genomes of *C. trachomatis* and *P. amoebophilia* code for a total of 917 and 2023 proteins, respectively. 224 out of 917 proteins were unique to *C. trachomatis*; 1129 out of 2023 proteins were unique to *P. amoebophilia*. Between the species 602 putative orthologs, 30 *C. trachomatis* and 2042 *P. amoebophilia* putative inparalogs and 18 putative coorthologs were found (as defined in Mendivil Ramos and Ferrier [16]; in our study, predicted on the basis of sequence similarity). A total of 741 sequence similarity-based protein families were predicted (Table 1). Protein family membership, with families numbered arbitrarily, is given in Additional file 1. Sequence alignment was performed using both the BLOSUM62 and BLOSUM45 substitution matrices, with only minor differences in results (Table 1; Additional files 2, 3, 4 and 5). Results based on BLOSUM62 were used for further investigation (Additional file 1).

**Table 1:** Analysis of protein families predicted across the genome-wide protein sets of *C. trachomatis* and *P. amoebophila*

Protein relationship	Number of protein families using BLOSUM62	Number of protein sequences using BLOSUM62	Number of protein families using BLOSUM45	Number of protein sequences using BLOSUM45
Single in both species	590	1180	586	1172
Unique to *C. trachomatis*	9	28	9	25
Unique to *P. amoebophila*	132	448	132	443
Single in *C. trachomatis* but multiple in *P. amoebophila*	8	28	7	25
Single in *P. amoebophila* but multiple in *C. trachomatis*	1	6	1	6
Multiple in both species	1	6	1	6

**Fig. 1:**
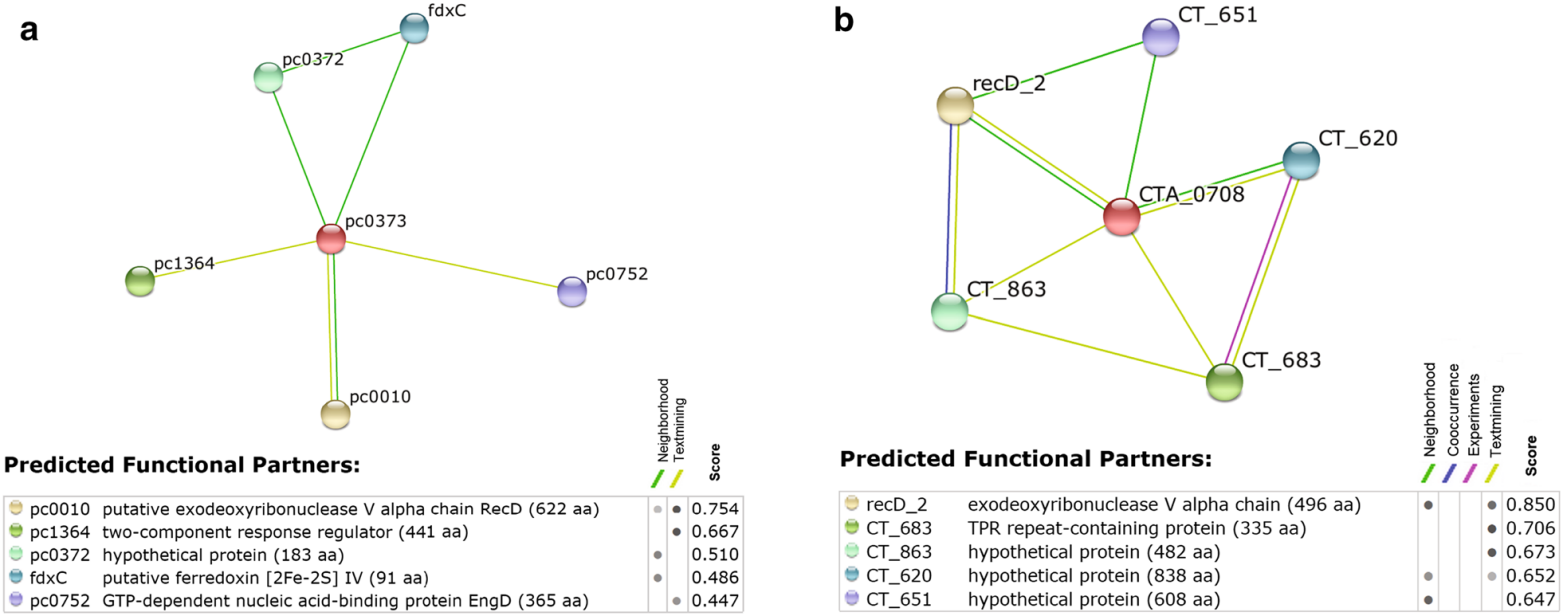
Predicting functional interactions of unannotated proteins. To further investigate the function of the protein family whose members were all unannotated, Group 40 (Additional file 1), functional interactions were investigated using the STRING database. It was found that *P. amoebophilia* Q6MEA2 (**a** STRING ID pc0373) and *C. trachomatis* Q3KL42 (**b** STRING ID CTA_0708) both interact with the (putative) exodeoxyribonuclease V alpha chain with a high confidence score. Each query protein is in the centre of the interaction web and is coloured *red*. *Grey dots* in the key represent strength of evidence (darker is stronger). The sum of each distinct evidence type was used to generate the total score

Protein families unique to *P. amoebophilia* included F-boxes and multiple transposases which catalyse movement of short DNA sections. Additionally, compared to single genes within *C. trachomatis*, multiple copies of virulence plasmid integrases, chaperonins, heavy metal transporters and putative antibiotic transporters were present (Table 2). Unique to *C. trachomatis* were five families of outer-membrane proteins, implicated in host evasion strategies, in addition to type III secretion system effectors. Present in multiple copies within both species were tyrosine transporters, indicating their essential function across the species as they lack the genes required for tyrosine biosynthesis (Table 2). Finally the functional interactions of some unidentified or hypothetical proteins were predicted using data from genomic context, co-expression and text-mining using STRING version 9.1 [17] (Fig. 1). We regard these predicted interactions as a basis for further study rather than a definitive result, and indeed many of these interactions are absent from a more recent version of STRING. Despite such uncertainties, the appearance of a (putative) exodoxyribose chain in both sets of interactors is suggestive.

**Table 2:** Differences in proteins produced, excluding shared single copy proteins

Protein relationship	Group number	Protein name
Unique to *P. amoebophila* *1	1	F-box
	2	Transposases
	3	Putative tetratricopeptide repeat protein
	4	Sel1 repeat protein4
	5	Transposases
Unique to *C. trachomatis*	10	Polymorphic outer membrane protein
	16	*2 Effector from type III secretion system
	70	Polymorphic outer membrane protein
	71	Hypothetical membrane associated protein
	72	Hypothetical membrane associated protein
	148	Deubiquitinase and deneddylase
	149	Biotin synthase
	150	*3 Threonine-rich GPI-anchored glycoprotein
	151	Outer membrane proteins
Single in *C. trachomatis* but multiple in *P. amoebophila*	11	Virulence plasmid integrases
	18	Low calcium response proteins
	19	Pb, Cd, Zn and Hg transporting ATPases
	36	Excinuclease ABC subunit A
	38	Chaperonins
	39	Putative antibiotic transporter
	40	*4
	41	Nucleoside diphosphate kinases
Single in *P. amoebophila* but multiple in *C. trachomatis*	9	Phosphatidylcholine-hydrolyzing phospholipase D (PLD) family
Multiple in both species	8	Tyrosine-specific transport protein

Protein families are uniquely identified by arbitrary group numbers, whose member proteins’ accession numbers are given in Additional file 1. For notes numbered *2 to *4, see Table 3. *1 In this category, only the largest five groups are shown. All proteins within these five groups were putative and uncharacterised, probable protein function was obtained by finding homologs on UniProtKB with >50 % sequence identity. For group three, although no homologs were found with >50 % sequence identity, it is possible that they are tetratricopeptide proteins as all within this group showed >30 % sequence identity to various tetratricopeptide proteins

**Table 3:** Putative, homology-based characterisation of proteins in Table 2

Note (asterisk)	Possible homolog	Species	Identity (%)	Additional comments
2	Effector from type III secretion system	*Chlamydia muridarum*	73	86 % positives
3	Threonine-rich GPI-anchored glycoprotein	*Chlamydia trachomatis*	80	84 % positives
4	Unknown	N/A	N/A	No homologs found; no secondary structure elements found; increased disorder at each terminal

This investigation builds upon the chlamydial comparison by Horn et al. [12], who identified proteins of interest such as virulence factors, transposases and tyrosine transporters. We quantify the differences in copy number in addition to suggesting roles for unidentified proteins.

## Discussion and conclusions

The scope of the study is limited. Where a protein is unique to one or other species in the pair, for example, it may be more widely distributed (among species not included in our study). Also, the direction of gain or loss of copies or families cannot be determined from a study of two species alone, but would require comparison of the gene or protein family phylogeny with the species phylogeny (e.g. [18]). However, where copy number varies within the pair of species study, this may indicate useful directions for future research.

Variation between the proteomes of *P. amoebophila* and *C. trachomatis* was expected due to differing host specificity. Our analysis identified protein families: unique to *C. trachomatis;* containing multiple members in *P. amoebophila* with one member in *C. trachomatis*; containing multiple members in *C. trachomatis* with one member in *P. amoebophila*; and one family with multiple members in both species. The latter family, with two members in *C. trachomatis* and four in *P. amoebophila,* consists of tyrosine-specific transport proteins.

To investigate the importance of the tyrosine transport proteins, other *Chlamydia* species should be investigated for the presence or absence of this protein family. If present in multiple copies across all *Chlamydia*, it could serve as a starting-point for development of a universal drug active across *Chlamydia*. A possible basis of substrate design would be a tyrosine analogue which binds irreversibly to *Chlamydia* tyrosine transporters only, and thus inactivates the transporter. This would be similar to the mechanisms of various NRTI class antiviral drugs that are nucleotide homologues e.g. AZT [19]. By targeting a whole protein family that is shared between species, any drug developed could act across the whole phylum.

Proteins unique to one species included various outermembrane proteins unique to *C. trachomatis* and multiple transposases unique to *P. amoebophila*. The abundance of transposases can account for the extensive genome rearrangement observed in *P. amoebophila* [14]. Further investigation into unique *P. amoebophila* proteins could reveal novel host-parasite interactions, such as why it causes apoptosis in human HEp-2 cells only when metabolically active [14]. Additionally, the presence of multiple polymorphic outer membrane proteins in *C. trachomatis* could be a mechanism of host immune system avoidance, especially during initial infection [20]. The use of STRING to predicted physical and functional partners could be applied to many of the groups where no known homology to any other sequence was found. Furthermore, the abundance of unknown protein families is an obstacle to understanding the host-parasite relationship. Characterisation of these unknown families would prove insightful to model many other bacterial endosymbiotic pathogens.

Although our study does not compare in depth the four major families of the Chlamidiae—as was done in [21]—it does provide an insight into the genetic and biological differences between human pathogenic chlamydia and the newly discovered endosymbiont. It also acts as a proof of concept, showing that the use of a low-cost Raspberry Pi computer in comparing genome-wide protein sets is successful in a bioinformatics research setting. The Raspberry Pi proved unproblematic for running BLAST, OrthoMCL and associated software and post-processing Perl scripts. Because of slow rendering of Web pages on the Raspberry Pi Model B, for convenience a desktop computer was used for Web access to the STRING database (see “Methods”, below). However, the newer Raspberry Pi version 2, not used in our study, would display Web pages faster [22]. One might also bypass the necessity of using the Web, by storing a local copy of STRING. This would be particularly useful in areas without Internet access.

The potential use for the Raspberry Pi or similar equipment in poor or isolated regions, as a tool to help identify pathogens, should be further investigated [10]. Applications for low-cost, Raspberry Pi-based comparisons of moderate numbers of genomes could include rapid mutation identification for viruses in rural areas and quick identification of crop moulds or pests in areas of famine.

There is also potential to democratize bioinformatics as a subject. Bioinformatics has abundant free software and sequence data, as used in our study and many others. These provide an exceptional starting point for democratization, but are not sufficient. Traditional barriers to wider uptake of bioinformatics include the cost of hardware. This barrier is addressed directly by relatively powerful, low-cost computers, including the Raspberry Pi. A persisting barrier is a lack of training [23, 24]. Free bioinformatics educational materials and programmes are increasing opportunities for training (e.g. [5, 25, 26]; for further references see [27]). With removal of these remaining barriers, we predict the expansion of bioinformatics research, by amateurs as well as students and professionals, including in low-income countries. We refer to this vision as ‘pervasive bioinformatics’, a concept which exists in the literature [28]—but is, itself, not yet pervasive.

## Methods

Bioinformatics software was run on a Raspberry Pi Model B with 521 GB RAM, under the 4273*π* variant of the Raspbian GNU/Linux operating system [5]. Genome-wide protein sets for *C. trachomatis* A/HAR-13 and Candidatus *P. amoebophila* UWE25 were downloaded from the Ensembl Genomes database (http://ensemblgenomes.org) [29] (Additional files 6 and 7). Sequence similarity-based protein families were predicted using MCL [30] and OrthoMCL [31] with default settings to post-process results of BLASTP sequence similarity searches [32]. Separate predictions were made, based on the BLOSUM62 (Additional files 2, 3 and 8) and on the BLOSUM45 substitution matrix (Additional files 4, 5 and 9). As no major differences were observed between the results (Table 1), groups obtained with BLOSUM62 were used for further analyses. Groups were counted and classified (Table 2) using custom Perl scripts (Additional files 10 and 11). Counts were verified using scripts written independently [11].

Protein functions were found either by manually integrating protein names from their Fasta headers, or by homology-based transfer of functional information from the UniProtKB database [33]. The five largest families unique to *P. amoebophila* were also analysed. Findings are presented in Table 2. An asterisk (*) indicates families where the majority were uncharacterised proteins in the *P. amoebophila* or *C. trachomatis* protein set, whose names were obtained from homology according to the following procedure. If the majority of the group were putative uncharacterised proteins, the first three protein IDs within the group text file were used as queries in BLASTP searches of UniProtKB [33]. If the three proteins had homologues similar in function it is assumed that the uncharacterised proteins also had the same function. If no homologues were found for a particular sequence, then the next protein in the group was investigated until a triplicate consensus was reached. In one case, Group 40 (Table 2), no homologues were found using BLAST. To predict protein function it was submitted to the STRING database, which contains data from genomic context, high throughput experiments and co-expression, using a desktop computer. To simplify the network diagram, only the five highest-scoring direct interactors are reported (Fig. 1).

## Additional files

10.1186/s13104-015-1476-2 Group Information. Contains the total number of proteins present in a group, how many copies exist between each species, the protein accession and name.
10.1186/s13104-015-1476-2 Protein Families (62). Lists OrthoMCL ortholog groups when using a BLOSUM62 matrix.
10.1186/s13104-015-1476-2 Group Counting Results (62). Contains the group IDs of families classified. Protein groups were initially generated using a BLOSUM62 matrix.
10.1186/s13104-015-1476-2 Protein Families (45). Lists OrthoMCLortholog groups when using a BLOSUM45 matrix.
10.1186/s13104-015-1476-2 Group Counting Results (45). Contains the group IDs of families classified. Protein groups were initially generated using a BLOSUM45 matrix.
10.1186/s13104-015-1476-2 *Chlamydia trachomatis* genome. The genome-wide protein set of *Chlamydia trachomatis*.
10.1186/s13104-015-1476-2 *Protochlamydia amoebophila* genome. The genome-wide protein set of Protochlamydia amoebophila.
10.1186/s13104-015-1476-2 Additional OrthoMCL output (62). Results based on pairwise alignment between the two species when using a BLOSUM62 matrix.
10.1186/s13104-015-1476-2 Additional OrthoMCL output (45). Based on pairwise alignment between the two species when using a BLOSUM45 matrix.
10.1186/s13104-015-1476-2 Group Counting Script. Script was used to count the unique groups of proteins, as well as identifying unique or shared groups.
10.1186/s13104-015-1476-2 Protein Function Identification Script. Sequentially reads each row of orthologs within an OrthoMCL group, extracts the protein name then outputs it.

## Abbreviations

AZT: azidothymidine
BLOSUM: blocks substitution matrix
*C. trachomatis*: *Chlamydia trachomatis*
*P. amoebophila*: Candidatus *Protochlamydia amoebophila*
RAM: random access memory
USB: universal serial bus

## References

[1] Raspberry Pi. Teach, learn and make with Raspberry Pi. http://www.raspberrypi.org.

[2] Krill P, Evenstad L. Raspberry Pi alternatives: 9 single-board computers for geeks. Computerworld UK. 2015. http://www.computerworlduk.com/galleries/it-vendors/raspberry-pi-alternatives-9-single-board-computers-geeks-3544497.

[3] BBC News. The Raspberry Pi computer goes on general sale. http://www.bbc.co.uk/news/technology-17190918.

[4] Collins K. Raspberry Pi is UK’s best selling computer. Wired. 2015. http://www.wired.co.uk/news/archive/2015-02/18/raspberry-pi-5-million.

[5] Barker D, Ferrier DEK, Holland PWH, Mitchell JBO, Plaisier H, Ritchie MG, Smart SD (2013). 4273*π*: bioinformatics education on low cost ARM hardware. BMC Bioinform.

[6] Pereira A, Atri M, Rogalla P, Huynh T, O’Malley ME (2014). Assessment of feasibility of running RSNA’s MIRC on a Raspberry Pi: a cost-effective solution for teaching files in radiology. Int J Comput Assist Radiol Surg.

[7] Leonard J. Of Raspberry Pi and CSI: how portable DNA analysis tools are helping police forensics, agriculture and medicine. Computing. 2015. http://www.computing.co.uk/ctg/analysis/2406922/of-raspberry-pi-and-csi-how-portable-dna-analysis-tools-are-helping-police-forensics-agriculture-and-medicine.

[8] Collet G, Rizk G, Chikhi R, Lavenier D. 2013. MINIA on Raspberry Pi—assembling a 100 Mbp genome on a credit card sized computer. http://f1000research.com/posters/1093759.

[9] Tso FP, White DR, Jouet S, Singer J, Pezaros DP. The Glasgow Raspberry Pi Cloud: a scale model for cloud computing infrastructures. In: IEEE 33rd international conference on distributed computing system workshops (ICDCSW) 2013, pp. 108–112.

[10] Ricke WF, Rasco DA (2014). Bacterial genome sequencing in the clinic: bioinformatic challenges and solutions. Nat Rev Genet.

[11] Wregglesworth K, Barker D. A comparison of the protein-coding genomes of two green sulphur bacteria, *Chlorobium tepidum* TLS and *Pelodictyon phaeoclathratiforme* BU-1. BMC Res Notes. Submitted. 10.1186/s13104-015-1535-8 PMC4606965 26467441

[12] Horn M, Collingro A, Schmitz-Esser S, Beier CL, Purkhold U, Fartmann B, Brandt P, Nyakatura GJ, Droege M, Frishman D, Rattei T, Mewes H-W, Wagner M (2004). Illuminating the evolutionary history of Chlamydiae. Science.

[13] Kalman S, Mitchell W, Marathe R, Lammel C, Fan J, Hyman RW, Olinger L, Grimwood J, Davis RW, Stephens RS (1999). Comparative genomes of *Chlamydia pneumoniae* and *C. trachomatis*. Nat Genet.

[14] Ito A, Matsuo J, Nakamura S, Yoshida A, Okude M, Hayashi Y, Sakai H, Yoshida M, Takahashi K, Yamaguchi H (2012). Amoebal endosymbiont *Protochlamydia* induces apoptosis to human immortal HEp-2 cells. PLoS One.

[15] Iliffe-Lee ER, McClarty G (2000). Regulation of carbon metabolism in *Chlamydia trachomatis*. Mol Microbiol.

[16] Mendivil Ramos O, Ferrier D. Mechanisms of gene duplication and translocation and progress towards understanding their relative contributions to animal genome evolution. Int J Evol Biol. 2012:846421. doi:10.1155/2012/846421. 10.1155/2012/846421 PMC3420103 22919542

[17] Franceschini A, Szklarczyk D, Frankild S, Kuhn M, Simonovic M, Roth A, Lin J, Minguez P, Bork P, von Mering C, Jensen LJ (2013). STRING v9.1: protein–protein interaction networks, with increased coverage and integration. Nucleic Acids Res.

[18] Stolzer M, Lai H, Xu M, Sathaye D, Vernot B, Durand D (2012). Inferring duplications, losses, transfers and incomplete lineage sorting with nonbinary species trees. Bioinformatics.

[19] Cihlar T, Ray AS (2010). Nucleoside and nucleotide HIV reverse transcriptase inhibitors: 25 years after zidovudine. Antiviral Res.

[20] Janeway CA, Travers P, Walport M, Shlomchik MJ. Evolution of the innate immune system. In: Immunobiology. 5th ed. New York: Garland Science; 2001.

[21] Collingro A, Tischler P, Weinmaier T, Penz T, Heinz E, Brunham RC, Read TD, Bavoil PM, Sachse K, Kahane S, Friedman MG, Rattei T, Myers GSA, Horn M. Unity in variety—the pan-genome of the *Chlamydiae*. Mol Biol Evol. 2011;28:3253–3270. 10.1093/molbev/msr161 PMC3247790 21690563

[22] Barisione M. WebKit on the new Raspberry Pi 2. 2015. http://blog.barisione.org/2015-02/webkit-rpi2.

[23] Lyantagaye SL (2013). Current status and future perspectives of bioinformatics in Tanzania. Tanzan J Sci.

[24] Tastan Bishop Ö, Adebiyi EF, Alzohairy AM, Everett D, Ghedira K, Ghouila A, Kumuthini J, Mulder NJ, Panji S, Patterton H-G (2015). Bioinformatics education—perspectives and challenges out of Africa. Brief Bioinform.

[25] Marques I, Almeida P, Alves R, João Dias M, Godinho A, Pereira-Leal JB (2014). Bioinformatics projects supporting life-sciences learning in high schools. PLoS Comput Biol.

[26] Corpas M, Jimenez RC, Bongcam-Rudloff E, Budd A, Brazas MD, Fernandes PL, Gaeta B, van Gelder C, Korpelainen E, Lewitter F, McGrath A, MacLean D, Palagi PM, Rother K, Taylor J, Via A, Watson M, Schneider MV, Attwood TK (2015). The GOBLET training portal: a global repository of bioinformatics training materials, courses and trainers. Bioinformatics.

[27] Barker D, Alderson RG, McDonagh JL, Plaisier H, Comrie M, Duncan L, Muirhead G, Sweeney S. University-level practical activities in bioinformatics benefit voluntary groups of pupils in the last two years of school. Int J STEM Educ. Submitted.

[28] Almeida JS, Iriabho EE, Gorrepati VL, Wilkinson SR, Grüneberg A, Robbins DE, Hackney JR (2012). ImageJS: personalized, participated, pervasive, and reproducible image bioinformatics in the web browser. J Pathol Inform..

[29] Cunningham F (2015). Ensembl 2015. Nucleic Acids Res.

[30] van Dongen S (2000). A cluster algorithm for graphs. Rep Inform Syst.

[31] Li L, Stoeckert CJ, Roos DS (2013). OrthoMCL: identification of ortholog groups for eukaryotic genomes. Genome Res.

[32] Altschul SF, Madden TL, Schäffer AA, Zhang J, Zhang Z, Miller W, Lipman DJ (1997). Gapped BLAST and PSI-BLAST: a new generation of protein database search programs. Nucleic Acids Res.

[33] The UniProt Consortium (2015). UniProt: a hub for protein information. Nucleic Acids Res.

